# Revisiting HIV-1 uncoating

**DOI:** 10.1186/1742-4690-7-96

**Published:** 2010-11-17

**Authors:** Nathalie Arhel

**Affiliations:** 1 Department of Virology, URA3015, Institut Pasteur, 25-28 rue du Dr. Roux, 75015 Paris, France

## Abstract

HIV uncoating is defined as the loss of viral capsid that occurs within the cytoplasm of infected cells before entry of the viral genome into the nucleus. It is an obligatory step of HIV-1 early infection and accompanies the transition between reverse transcription complexes (RTCs), in which reverse transcription occurs, and pre-integration complexes (PICs), which are competent to integrate into the host genome. The study of the nature and timing of HIV-1 uncoating has been paved with difficulties, particularly as a result of the vulnerability of the capsid assembly to experimental manipulation. Nevertheless, recent studies of capsid structure, retroviral restriction and mechanisms of nuclear import, as well as the recent expansion of technical advances in genome-wide studies and cell imagery approaches, have substantially changed our understanding of HIV uncoating. Although early work suggested that uncoating occurs immediately following viral entry in the cell, thus attributing a trivial role for the capsid in infected cells, recent data suggest that uncoating occurs several hours later and that capsid has an all-important role in the cell that it infects: for transport towards the nucleus, reverse transcription and nuclear import. Knowing that uncoating occurs at a later stage suggests that the viral capsid interacts extensively with the cytoskeleton and other cytoplasmic components during its transport to the nucleus, which leads to a considerable reassessment of our efforts to identify potential therapeutic targets for HIV therapy. This review discusses our current understanding of HIV uncoating, the functional interplay between infectivity and timely uncoating, as well as exposing the appropriate methods to study uncoating and addressing the many questions that remain unanswered.

## Structure of mature HIV-1 capsid and its importance at early stages of infection

**Figure 1:**
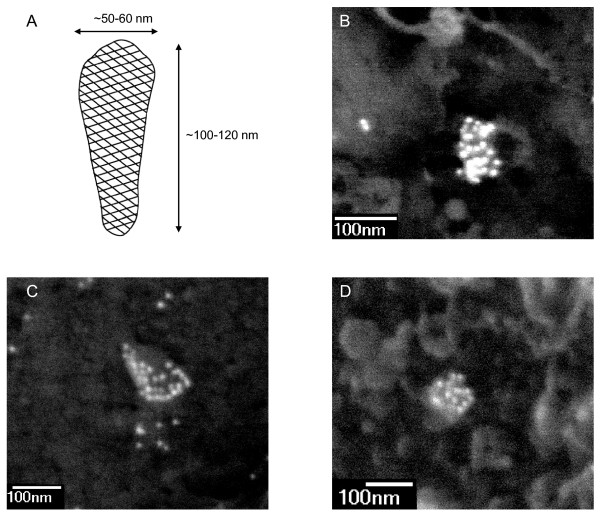
**Scanning electron microscopy imaging of HIV-1 capsids in the cytoplasm and at the nuclear membrane of infected cells**. **(A) **Schematic representation of the mature HIV-1 capsid shell. The HIV-1 capsid is an assembly of approximately 1,500 CA monomers arranged into a hexagonal array of hexamers. Dimensions are derived from microscopy observations of mature virions or isolated cores. **(B-D) **Images show the backscattered gold signal corresponding to specific labelling with a mouse monoclonal anti-p24 antibody (183-H12-5C AIDS Reagent Program) followed with goat anti-mouse IgG H&L conjugated 10 nm gold (British Biocell International) in HIV-1 infected P4-CCR5 cells. HIV-1 capsids are typically conical- or cylindrical-shaped, ca 100-150 nm long, and heavily labelled with 10-30 immunogold particles. The bulk of antibodies likely induces some distortions in size and shape of capsids. In panels B and D, capsids are located at the nuclear membrane: nuclear pore complexes appear as bright rings with dark lumen.

The mature HIV-1 capsid, also called HIV-1 core, is a highly organised macromolecular assembly, formed within newly released virions upon proteolytic cleavage of the precursor p55Gag polyprotein by the viral protease which generates the cleavage product CA (also called capsid or p24). Rather confusingly, the term capsid refers both to the conical multimeric structure and to the CA monomers that constitute the cone. Therefore, to avoid all confusion, the terms "capsid" and "core" are preferred for reference to the conical structure and monomers are referred to as "CA". Negative staining and cryo-electron microscopy of authentic mature particles or isolated mature HIV-1 cores reveal that capsids have an intriguing conical shape, with a relatively consistent length of 100-120 nm [1-4] (Figure 1). The diameter of the wide end of the capsid cone (50-60 nm) and the angle at the tip of the cone (18-24°) may vary and lead to capsids with apparent heterogeneity of shape (bullet shape, cylindrical forms).

The intrinsic properties of the HIV-1 capsid, such as its poor stability or asymmetry, have made it particularly difficult to explore the detailed structure of mature cores isolated from disrupted virions. However, recombinant CA can spontaneously assemble *in vitro *into cones and structures analogous to authentic HIV-1 capsids [5] and much of the valuable information we have on the shape and underlying molecular structures of the capsid derive from core-like structures obtained from *in vitro *CA assembly reactions. These have shown that despite its macromolecular asymmetry, the HIV-1 capsid is assembled with a high degree of organisation as a fullerene cone, a structure with hexagonal lattice symmetry that is capped at both ends [5,6]. The HIV-1 capsid is made up of ca. 1,500 CA monomers, which assemble into 250 hexameric rings through NTD-NTD (N-terminal domain) interactions, which are themselves linked into a hexagonal lattice through CTD-CTD (C-terminal domain) interactions [7,8]. The hexagonal lattice is curved into a cone through subunit mobility [8] and is capped by exactly 12 pentameric rings, 7 at the wide end and 5 at the narrow end of the cone [5].

The capsid contains the viral genome (two single stranded RNA molecules), some viral proteins (CA, nucleocapsid (NC), reverse transcriptase (RT), integrase (IN), Vpr) and numerous cellular proteins, such as Cyclophilin A and APOBEC3G [9]. Its main function is to organise and contain the viral genome for optimal delivery in target cells and efficient reverse transcription, which together contribute to effective replication in the new host cell. The capsid cone is absolutely essential for infection since mutations of protease cleavage sites in Gag, or inhibitors of Gag processing, produce immature virions and abolish HIV-1 infectivity [10-13]. Furthermore, point mutations that lead to hyperstable or unstable capsids, or to capsids with aberrant morphologies, lead almost systematically to reduced infectivity [14], indicating that the shape and stability of the capsid are also critical for HIV-1 infectivity.

## The necessity of uncoating for HIV-1 and other lentiviruses

HIV-1 and other lentiviruses are unique among orthoretroviruses in their ability to replicate efficiently in metabolically active non-dividing cells [15,16] as a result of the active nuclear import of their genome across the nuclear membrane of interphasic nuclei [17]. Retroviruses such as the Murine Leukaemia Virus (MLV) gain access to the nuclear chromatin following the disassembly of the nuclear membrane that occurs during mitosis [18]. For such retroviruses, evidence suggests that the viral capsid accompanies the viral genome into the nuclear compartment and participates in interaction with the chromatin [19] indicating that uncoating is not required prior to nuclear import.

HIV and other lentiviruses enter the nuclei via the nuclear pore; and, although commonly assumed, it is by no means certain that they can use an alternative route of entry during mitosis. Indeed, the replication of certain lentiviruses (such as EIAV, CAEV and VISNA) is entirely limited to macrophages, which do not divide. In the case of HIV-1, which infects cycling CD4+ T cells in addition to macrophages, a mitosis-independent nuclear import in cycling cells has been reported [20]. Furthermore, a genome-wide RNA interference-based screen comparing HIV-1 and MLV infections identified unique nuclear import factors for HIV-1 even though the study was carried out in cycling cells [21]. In addition, HIV-1 mutants with a nuclear import defect in cell cycle-arrested cells often maintain this defect in cycling cells [22-25]. Finally, the assumption that HIV-1 might passively gain access to the chromatin upon mitosis, if based on the belief that cytoplasmic and nuclear contents mix homogeneously throughout mitosis, is not valid. Indeed, evidence suggests that mitotic cells maintain spatial information through gradients, such as the RanGTP gradient that surrounds chromatin [26,27]. Taken together, it is probable that HIV-1 enters nuclei only through the nuclear pore whether cells divide or not.

The dependency of lentiviruses on non-dividing cells for *in vivo *transmission and persistence and the resulting necessity to enter the nucleus through the nuclear pore impose an uncoating step because the diameter of the viral capsid (up to 60 nm wide) exceeds that of the nuclear pore (~30 nm). Consistent with this notion, previous work has reported a substantial difference in mass between cytoplasmic and nuclear HIV-1 complexes [28,29] and the absence of CA within pre-integration complexes [30,31].

## Where and when does uncoating occur?

Although most agree that uncoating occurs after fusion-dependent entry in the cytoplasm and before nuclear import, the field remains divided as to the precise moment and location for this event. Indeed, the extent of the role of HIV-1 capsid at early stages of infection is still a matter of debate. In a first model, the viral capsid is disassembled close to the plasma membrane immediately following fusion into the cytoplasm and most CA is dissociated from the HIV-1 nucleoprotein complex [32-35]. Uncoating is required for formation of the reverse transcription complex (RTC) and is likely triggered by the sudden change in environment in which the viral complex finds itself, or possibly by the loss of high concentrations of free CA present in virions and responsible for maintaining metastable cores [36]. In this model, the absence of significant amounts of CA within intracellular HIV-1 complexes soon after inoculation [28,29,37-39] and the inability to detect capsids in the cytoplasm of infected cells using transmission electron microscopy (TEM) [40] led to the conclusion that the primary function of HIV-1 capsid is to deliver the viral genome into the cytoplasm, after which it can and must be discarded for productive infection to proceed, although it is not excluded that initial disassembly is partial [32-35].

A second model proposes that capsid remains intact for some time post-entry, at least for the initiation of reverse transcription, and that uncoating occurs gradually during transport towards the nucleus and reverse transcription [41]. In this model, uncoating is promoted in response to multiple successive changes in the cellular environment, sequential contact with different cell factors, and through the molecular rearrangements that accompany reverse transcription, thus triggering progressive or stepwise conformational changes and disassembly. In support of this hypothesis are studies that report a broad range of different sizes and shapes for cytoplasmic HIV-1, both greater and smaller than mature extracellular cores This suggests a complex series of transformations accompanying reverse transcription and transport to the nucleus [39,41-43] (although it cannot be excluded that the observed variations are due to the preparation or isolation protocol [44]), immunofluorescent  microscopy showing association of CA with RTCs [42], and the demonstration that capsids with increased or decreased core stability has impaired reverse transcription [14].

A third model, which we favour, proposes that capsids remain intact until HIV-1 incoming complexes reach the nuclear membrane and that uncoating occurs at the nuclear pore upon completion of reverse transcription. In this model, the HIV-1 capsid is all-important for maintaining a high stoichiometry of HIV-1 reverse transcriptase enzyme relative to the viral genome during reverse transcription to counteract its tendency to dissociate from its template [45], since dilution of reverse transcriptase in the cytoplasm would lead to highly ineffective reverse transcription. While it organises the HIV-1 viral genome and proteins, it offers no impermeable environment from small macromolecules of the cytoplasm: the capsid lattice is an open structure, with inter-ring spacings of up to 10 nm [6], which allow small macromolecules, such as nucleotide triphosphates and indeed reverse transcriptase inhibitors, to access the reverse transcription complex in the cytoplasm of infected cells. While the first model proposes that uncoating (at the plasma membrane) is required to trigger viral reverse transcription, this third model suggests that it is the successful completion of reverse transcription (at the nuclear pore) that triggers uncoating.

Recent evidence suggests that the integrity and timely disassembly of the HIV-1 capsid are essential for routing to the nuclear compartment, reverse transcription and successful nuclear import [14,29,46-48]. For this reason, there is an increasing appeal to determine up to which point the viral capsid is required for infection and at which point in space and time it is disassembled.

## Uncoating accompanies the conversion of RTCs into pre-integration complexes (PICs)

**Figure 2:**
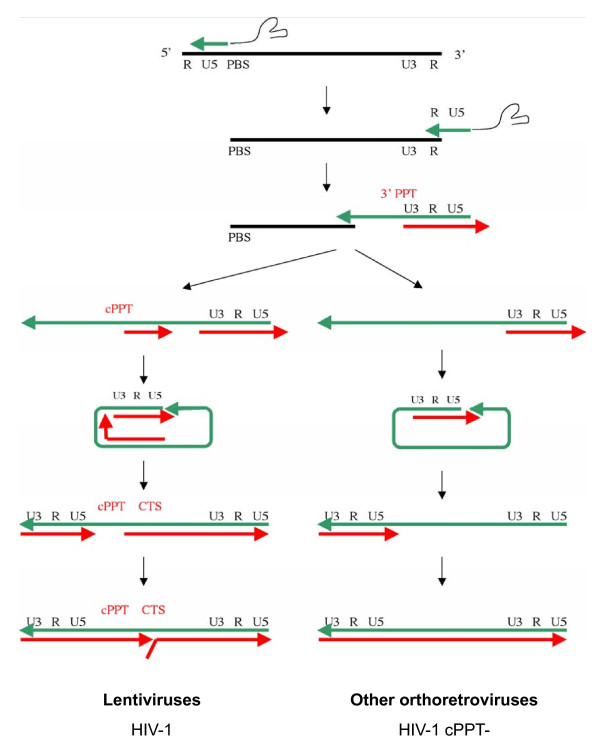
**Schematic representation of reverse transcription in lentiviruses and other orthoretroviruses (such as MLV)**. The conversion of the single-strand RNA genome (represented as a black line) into double stranded DNA genome (at the bottom of the diagram) is the hallmark of retroviruses. Reverse transcription is initiated by the synthesis of minus-strand DNA (in green) at the PBS site (Primer Binding Site) at the 5' end of the RNA genome. The minus-strand strong-stop DNA thus synthesised is then transferred to the 3' end of the genome through complementarity with the R (Repeated) region of the LTR region (Long-Terminal Region) thus allowing synthesis of the minus-strand DNA to be completed. Minus strand DNA synthesis is accompanied by progressive degradation of the RNA matrix by the RNase H activity of reverse transcriptase. Two RNA sequences resist RNase degradation because they contain a unique PPT sequence and these serve as initiation sites for the plus-strand DNA. In all retroviruses, plus-strand DNA synthesis (in red) is initiated at the 3'PPT. In the case of lentiviruses, initiation also takes place at the cPPT. After a second strand transfer, plus-strand DNA synthesis proceeds to generate double-stranded DNA. In the case of lentiviruses, plus-strand initiation in two distinct sites leads to a displacement of the downstream strand over *ca *100 nucleotides, terminating at the CTS and thus generating a discrete strand displacement called the central DNA Flap.

In early replication, incoming HIV-1 is referred to either as an RTC or as a PIC. The literature is divided as to the nature and location in the cell of HIV-1 RTCs and PICs, and most importantly as to the presence or lack of capsid in these complexes. RTCs are simply defined as HIV-1 complexes that undergo reverse transcription, during which they convert their single-stranded positive RNA viral genome into double-stranded DNA [49,50] (Figure 2). The RTC genomes are thus either RNA or RNA-DNA intermediates of reverse transcription. In contrast, PICs no longer contain any RNA but only the double-stranded DNA. PICs are per definition integration-competent HIV-1 complexes and can integrate efficiently into a target DNA *in vitro *[51,52]. They are formed in the cytoplasm upon synthesis of full-length viral DNA and then translocate into the nucleus where they form the integrated provirus.

It is assumed that reverse transcription is triggered by the exposure of the viral complex to non-limiting deoxyribonucleotides in the cytoplasm [44]. Reverse transcription involves firstly the formation of the minus strand strong-stop DNA, a strand transfer event, and the synthesis of the minus strand DNA with concomitant degradation of the RNA template. In the HIV-1 genome, two polypurine tracts (PPT), the central PPT (cPPT) and 3' PPT, resist degradation by RNase H and serve as primers for synthesis of plus-strand DNA [49,53,54]. Reverse transcription proceeds with synthesis of plus-strand DNA, involves a second strand transfer event, and terminates at a central termination sequence (CTS) in the centre of the genome. The initiation of plus-strand synthesis at the cPPT, as well as the 3' PPT common to all retroviruses, leads to a discrete plus-strand displacement of *ca *100 nucleotides in the centre of the genome (Figure 2). The final product of HIV-1 reverse transcription is therefore a linear double-stranded DNA with a central DNA Flap [54]. The duration of reverse transcription varies according to the metabolic state of the cell and in the case of asynchronous infection. Full-length linear DNA may be detected as early as 4 h post-infection but reaches its peak at 8-12 h post-infection [22,55,56]. Upon DNA Flap formation and completion of reverse transcription, the viral complex becomes a PIC, competent for import into the nucleus and integration within the host cell chromatin.

In the PIC, the *~ *9.7 kb HIV-1 genome, which would per definition measure up to 3.3 μm, is compacted into a 56 nm diameter object [31], possibly by viral and/or cellular proteins that additionally could render it karyophilic for passage through the nuclear pore. Although the complete identification of PIC components remains elusive due to the difficulty to isolate PICs from infected cells, many viral and cellular factors have been identified as PIC components [34]. PICs are devoid of detectable CA proteins [30,31] and contain IN [30]. The presence of other viral proteins such as NC, matrix (MA), RT and Vpr is a source of debate [28-31,37]. Several cellular factors have also been shown to associate with HIV-1 PICs, such as the high mobility group protein HMG I(Y) [31] and LEDGF/p75 [57]. Although interaction of these cellular factors with HIV-1 PICs may occur in the cytoplasm, their role in HIV-1 infection becomes apparent in the nucleus where they may assist tethering of the PIC to the chromatin, determine integration site selection and assist integration [31,58,59].

Clearly, the transition between RTC and PIC is associated with uncoating, however the fragile nature of the HIV-1 capsid and the complexity of the early phases of HIV-1 infection have made it particularly difficult to pinpoint when this occurs.

## Experimental hurdles to studying the fate of HIV-1 capsids in newly infected cells

The most straightforward way to study uncoating in infected cells is to isolate RTCs at given time points post-infection using sedimentation velocity gradients and to probe for co-sedimentation of CA with the viral genome; or conversely to immunoprecipitate cellular extracts with anti-CA antibodies and probe for viral genome by PCR. Using these approaches, CA was not found to be substantially associated with the viral genome within the cytoplasm of infected cells, thus leading to the conclusion that the viral capsid is discarded from RTCs rapidly after cell entry [28,29,37-39]. However, the HIV-1 capsid is inherently unstable and disassembles readily in the presence of non-ionic detergents and upon ultracentrifugation [2,60]. Moreover the size, shape and components of isolated RTCs are highly dependent on the conditions used for isolation, particularly on the detergent and salt concentrations [44]. Therefore, it cannot be excluded that the complexes analysed by biochemical isolation approaches have in fact lost their capsid during the isolation procedure.

A further difficulty comes from the fact that most incoming viral complexes are more likely to be destined for degradation than on a productive pathway for infection since over 85% of viruses that have entered the cell do not form proviruses [61]. This is the case of viral particles that have entered by endocytosis [62] and of functional RTCs that are lost in the routing process towards the nucleus [42]. As a result, the majority of cytoplasmic RTCs isolated early after infection likely represent complexes that were damaged or engaged in a pathway of degradation at the time of isolation or observation.

In order to circumvent the need to isolate RTCs from infected cells, some groups have attempted to visualise HIV-1 capsids by *in situ *ultrastructural electron microscopy in infected cells. Generally speaking however, it is difficult to follow the fate of viral complexes inside the cytoplasm using morphological criteria in sections of electron microscopy (EM) [63]. Indeed, a single viral capsid would be almost impossible to distinguish from other cytoplasmic components unless it was fortuitously cut right along the length of the capsid, thus displaying its unusual conical morphology. Any other cut will lead to heterogeneous circular and ovoid structures. Furthermore, the visualisation of intracellular capsids within a 60 nm thick EM slice, which represents less than 1:50^th ^of the cell thickness, is per definition a rare event. Using this ultrastructural approach with TEM, few [47] or no [40] intact virus cores were observed in the cytoplasm of infected cells.

The intrinsic difficulty of studying HIV-1 uncoating has driven the development of alternative approaches. Using *in situ *immunohistochemical approaches, HIV-1 CA is readily detected throughout the cytoplasm of infected cells and co-localises with the viral genome [42,47]. Nevertheless, in order to demonstrate that this CA signal corresponds to capsid cores rather than soluble CA, immunolabelling of CA or detection of the viral genome must be combined with ultrastructural observations. This is especially difficult to achieve using TEM since preparations generally favour either ultrastructural observations or immunolabelling. One approach to overcome this involves detection of the viral DNA using *in situ *hybridisation with electron microscopy [64] without the usual protease treatment in order to preserve proteinaceous structures as much as possible. Using this approach, capsid shells could be detected around the viral genome but with weak intensity [47]. An alternative approach involves observing intracellular complexes, *in situ *via a scanning EM (SEM), in cells stripped of their plasma membrane [47,65]. This ultrastructural approach may be readily combined with immunolabelling and may enable the observation of intracellular HIV-1 capsids, identified by specific anti-CA labelling and a morphology similar to intact viral cores [47] (Figure 1).

To study uncoating in a quantitative manner, particulate (intact) capsids may be separated from soluble (monomeric) CA by ultracentrifugation of virions through a sucrose cushion overlaid with a low concentration of detergent [66]. This cell-free assay enabled to analyse the effects of mutations on capsid stability [14,67,68] and of reverse transcription on capsid integrity [47]. A variation of this assay enables the study of capsid uncoating in infected cells by carefully designed ultracentrifugation of cell lysates through a sucrose cushion, which separates cytosolic cores from soluble CA [69]. This fate-of-capsid assay has been used for example to establish a correlation between retroviral restriction and accelerated uncoating [69,70], to study capsid stability in infected cells [71], and quantify retroviral restriction potency and kinetics [72,73].

Given the complexity and fragile nature of the HIV-1 capsids, there is still a need for sensitive, specific and reliable assays for uncoating. No assay may be relied upon solely when interpreting uncoating events.

## Timely uncoating is key for HIV-1 reverse transcription, nuclear import and infectivity

### Lessons from retroviral restriction: premature uncoating leads to abortive infection

The tripartite motif 5-alpha (TRIM5α) protein is a dominant factor of intrinsic immunity that mediates cellular restriction against retroviral infections in a species-specific manner [74,75] and was originally discovered as a determinant of the resistance of monkey cells to HIV-1 infection [76]. Although the exact mechanisms that lead to virus inactivation by TRIM5α proteins remain unclear [77], it is known that TRIM5α targets intact retroviral capsids early in viral replication prior to reverse transcription, by interacting directly with these through its B30.2 (SPRY) C-terminal domain [69,78,79]. Interestingly, Fv1 restriction of MLV in mouse cells, although known to involve different mechanisms than TRIM5α, also targets capsid cores [80,81]. Retroviral restriction mechanisms are thought to have evolved in many species including primates as a result of evolutionary pressure exerted by continual exposure to retroviruses [77]. The startling evolutionary conservation of recognition of capsid cores as restriction mechanism suggests that their structure, composition and stability are key to retroviral infections.

**Figure 3:**
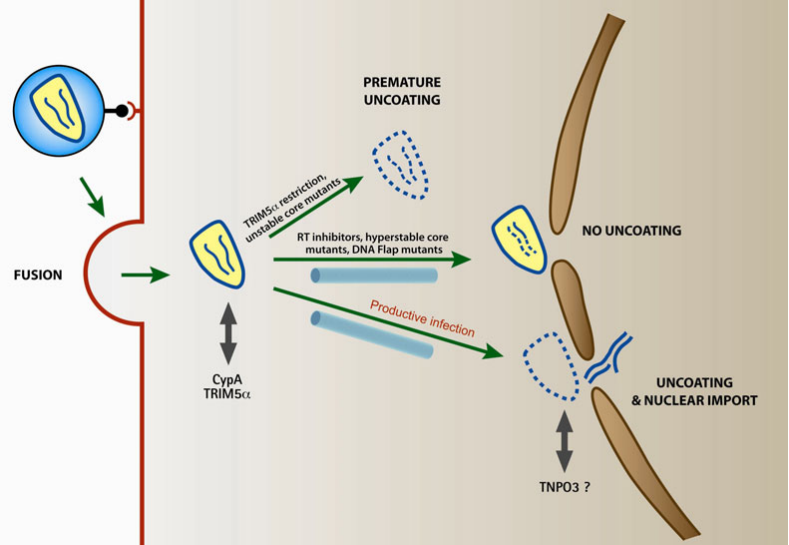
**Schematic representation of the fates of viral capsids in the cytoplasm of newly infected cells**. After entry into the cytoplasm, HIV-1 capsids that are on a path of productive infection remain intact and are transported towards the nucleus along the cytoskeleton. They uncoat at the nuclear membrane upon completion of reverse transcription. Premature uncoating, in the case of TRIM5α restriction or of unstable capsid mutants, leads to abortive infection. Similarly, compromised uncoating, in the case of incomplete reverse transcription or of hyperstable capsid mutants, also leads to a dead-end infection event.

In the case of restriction of HIV-1 in rhesus macaque cells, it was originally supposed that TRIM5α binding might inhibit the uncoating of the viral capsid [82,83], thought to be required for reverse transcription to initiate in the cytoplasm of newly infected cells. However, more recent work indicates that TRIM5α, quite on the contrary, promotes the rapid and premature disassembly of viral capsids thus abrogating productive reverse transcription [69,70,84] (Figure 3). These findings indicated for the first time that premature uncoating of HIV-1, far from being beneficial for initiation of reverse transcription and infection, is in fact detrimental to both and is the molecular cornerstone for potent species-specific retroviral restrictions. This suggests that the stability and integrity of HIV-1 capsids during the early steps of infection is key to effective replication. In support of this are findings that capsid mutants with either reduced or increased stability compromise almost systematically HIV-1 infection [14] (Figure 3), indicating that both too rapid and too slow uncoating are detrimental for HIV-1 infection.

### Lessons from reverse transcription: unsuccessful reverse transcription precludes uncoating

One hypothesis proposes that uncoating might occur in response to changes in viral nucleic acid nature and structure, rather than in changes in cellular environment. If this were the case, then inhibiting reverse transcription would be expected to arrest uncoating at a very defined and reproducible step. Strikingly, blocking reverse transcription by a reverse transcriptase inhibitor (Nevirapine) resulted in the accumulation of conical capsid cores in proximity to and at the nuclear membrane and nuclear pores at late time points post-infection [47]. These were formally identified as HIV-1 capsids based on specific anti-CA labelling and morphological criteria, and were shown to contain the viral genome using EM *in situ *hybridisation. An accumulation of intact capsids at the nuclear membrane was also observed in the case of inhibition of DNA Flap formation, the last chronological event of reverse transcription [47] (Figure 1). Therefore, although incoming HIV-1 capsids may undergo stepwise destabilisation during cell entry and cytoplasmic transport, these data suggest that HIV-1 uncoating is not progressive but occurs upon completion of reverse transcription. They also suggest that progression through reverse transcription and uncoating is independent of transport towards the nucleus.

### Lessons from nuclear import: timely uncoating underlies the ability of HIV-1 to infect non-dividing cells

The search for the determinants of nuclear import that allow HIV-1 and other lentiviruses to infect non-dividing cells is an active and controversial field of investigation [85]. Based on the search of nuclear localization sequences, a number of HIV-1 proteins have been proposed to contribute in a redundant manner to the karyophilic properties of the HIV-1 PIC but the actual participation of these proteins in HIV-1 genome nuclear import has been a matter of strong debate [85]. The integrase protein, which is tightly associated with PICs until the integration of the viral DNA into the host chromosomes, is karyophilic and may participate in HIV-1 nuclear import. The cis-acting sequences cPPT and CTS, which form the central DNA Flap during reverse transcription, have also been identified as determinants of HIV-1 genome nuclear import [22] and are as a result systematically inserted within lentiviral vectors to enhance gene transfer efficiencies.

Intriguingly, recent work based on capsid mutants or chimeras has introduced the existence of a functional link between the HIV-1 CA and nuclear import [46,48,86], underlying the importance of timely uncoating for nuclear import. Furthermore, the requirement of HIV-1 nuclear import for transportin-SR2 [87][88], also called TNPO3, has been mapped to the HIV-1 CA [89]. Although an interaction between HIV-1 capsid and TNPO3 remains to be demonstrated, a CA point mutation renders HIV-1 insensitive to TNPO3 knockdown [90]. Uncoating is necessary for passage through the nuclear pore, and HIV-1 complexes that fail to uncoat will accumulate at the cytoplasmic face of the nuclear membrane [47]. However, these data further suggest that HIV-1 CA may also be essential to mediate interaction with the nuclear pore, with transport proteins such as transportin-SR2, or with nucleoporins prior to uncoating.

## Conclusion: rethinking HIV-1 uncoating

Previous work led us to suppose that the HIV-1 capsid core, although all-essential for initial delivery into the cytoplasm, is then discarded immediately post-fusion to stimulate reverse transcription. Others propose that uncoating probably occurs gradually, possibly in response to multiple cellular cues such as interaction with cellular proteins or subcellular localisation, or viral cues such as the progress of reverse transcription. Although incoming capsids may undergo progressive destabilisation during their transport towards the nucleus, since hyperstable capsid mutants have impaired reverse transcription [14], recent independent experiments suggest that the position and timing of uncoating may in fact be tightly regulated and have a trigger. Uncoating must be neither too early nor too late in order to ensure productive infection (Figure 3).

Although the intricacies of HIV-1 uncoating - its timing, location and mechanism - are by no means resolved, recent work enables us to etch a possible model for the early steps of HIV-1 infection. Entry of HIV-1 into target cells delivers the intact capsid core into the cytoplasm and exposure of the viral nucleoprotein complex to non-limiting deoxyribonucleotides triggers reverse transcription. This likely occurs within the intact capsid core, which is essential for maintaining a high concentration of enzyme around the nucleic acid while being entirely permeable to the necessary deoxyribonucleotides. During reverse transcription, HIV-1 RTCs move rapidly toward the nuclear compartment, using microtubules then actin filaments to reach the nuclear pore [42,47]. Since transport to the nuclear pore (within minutes to 1-2 hours) is more rapid than reverse transcription (8-12 hours), it is likely that most viral DNA synthesis occurs within capsid cores docked at the nuclear pore. This implies that subcellular fractionation experiments that do not distinguish between nuclear membrane and nucleoplasm are in fact incapable of distinguishing nuclear from cytoplasmic HIV-1 complexes. Indeed, HIV-1 complexes docked at the nuclear membrane will appear in the nuclear fraction even though they are in fact in the cytoplasm. The presence of intact HIV-1 capsids at the nuclear membrane further implies that the most likely viral structure that interacts with the host cell during transport towards the nucleus is the capsid core. Using a yeast-two-hybrid screen and interaction assays with capsid cores, we identified several components of the microtubule and actin network as interaction partners for HIV-1 capsid and essential co-factors of HIV-1 infection (A. Becker, S. Munier, N. Arhel, unpublished data). Therefore, as well as being essential for reverse transcription, the capsid shell may also be key to bringing viral complexes to their site of replication.

One hypothesis brought forward is that the completion of reverse transcription and the formation of the central DNA Flap trigger or facilitate uncoating [47]. If this is the case, then the trigger for uncoating is not a cellular cue, as is the case for adenoviruses [91] or herpes simplex virus type 1 [92], but a viral signal. Concordant with this, uncoating can occur *in vitro *upon synthesis of full-length viral DNA by endogenous reverse transcription, suggesting that any cellular factors required for uncoating are present within HIV-1 virions [47]. Apart from the DNA Flap, other viral and cellular factors have been proposed to participate in uncoating, including IN [93], prolyl isomerases Pin1 [94] and Cyclophilin A [72], and cellular factors present in non-resting cells [95].

A further hypothesis is that uncoating occurs at the nuclear pore [47] and allows PICs to be imported into the nucleus. Consistent with this is the fact that CA may constitute a determinant of HIV-1 PIC nuclear import [46,48,86,89]. Premature uncoating, as in the case of TRIM5α restriction or unstable capsid mutants, leads to abortive infection. Similarly, complexes that fail to uncoat, such as hyperstable capsid mutants or in the case of inhibited reverse transcription, cannot be imported into the nucleus.

Much of previous work was interpreted in light of the assumption that HIV-1 uncoating occurred immediately post-fusion and the association of CA with intracellular HIV-1 was understood to be detrimental for HIV-1 infection. If we accept the premise that HIV-1 capsids uncoat at the nuclear pore upon completion of reverse transcription, our interpretation of data must be reversed: the loss of capsid cores after entry then corresponds to early degradation products of abortive complexes and the maintaining of intact capsids to complexes on the path of productive infection.

## Perspectives

The molecular mechanisms underlying the destabilisation and uncoating of HIV-1 in the cytoplasm of infected cells remain to be elucidated. Both cytoplasmic environment and major rearrangements of the RTC at the end of reverse transcription could contribute to the disassembly of capsids prior to nuclear import. Furthermore, the importance of timely uncoating for HIV-1 infection and the fact that the capsid is the target of evolutionary conserved anti-retroviral restriction mechanisms emphasise the interest to develop a new class of anti-retroviral drugs that either accelerate or entirely inhibit uncoating.

## Competing interests

The author declares that they have no competing interests.
